# Association between hypertension and oxidative balance score: data from National Health and Nutrition Examination Survey 2005–2018

**DOI:** 10.3389/fcvm.2025.1538095

**Published:** 2025-06-24

**Authors:** Linfeng Tao, Yanyou Zhou, Lifang Wu, Yue Zhu, Juntu Li, Chao Li, Yiyuan Pan, Jun Liu

**Affiliations:** ^1^ Department of Critical Care Medicine, The Affiliated Suzhou Hospital of Nanjing Medical University, Suzhou Municipal Hospital, Suzhou Clinical Medical Center of Critical Care Medicine, Gusu School of Nanjing Medical University, Suzhou, China; ^2^ Department of Critical Care Medicine, Kunshan Third People’s Hospital, Suzhou, China; ^3^ Department of Breast and Thyroid Surgery, The Affiliated Suzhou Hospital of Nanjing Medical University, Gusu School, Nanjing Medical University, Suzhou, China; ^4^ Department of Critical Care Medicine, Suzhou Municipal Hospital, Suzhou, China

**Keywords:** hypertension, oxidative stress, oxidative balance score, NHANES, OBS

## Abstract

**Background:**

Hypertension, a prevalent worldwide public health issue, can result in a wide range of illnesses. The notably association between oxidative stress and the onset of hypertension has been corroborated through diverse animal models. The oxidative balance score (OBS) served as a tool to evaluate the overall systemic status of oxidative stress, indicating that higher OBS scores corresponded to greater exposure to antioxidants. However, the exact correlation between OBS and hypertension is unclear. Therefore, we aimed to investigate whether adult OBS is attached to hypertension.

**Methods:**

There are 28,035 participants who were chosen from the National Health and Nutrition Examination Survey (NHANES) conducted between 2005 and 2018. The presence of hypertension was determined through a questionnaire. Twenty food and lifestyle parameters were used to score OBS. The connection between OBS and hypertension has been examined via weighted logistic regression and smoothing curves.

**Results:**

The percentage of people with hypertension stood at 41.72%. In comparison to the first quartile of OBS, the adjusted odds ratios for the highest OBS quartile and hypertension were 0.81 (95% CI: 0.70–0.93), with a *p*-value for trend of 0.002. Age was the factor most strongly linked with both in stratified analysis.

**Conclusions:**

OBS demonstrated a strong negative association with hypertension, particularly in the younger population (<60 years). These findings highlighted the importance of following an antioxidant-rich diet and lifestyle, which aids in hypertension prevention and appears to offer greater benefits to the younger age group.

## 1 Introduction

Hypertension is a prevalent cardiovascular disease in older adults. Over the past few years, hypertension has progressively grown to be a global health concern (1). From 1990–2019, the prevalence of hypertension doubled, with a total of approximately 1.2 billion adults global having hypertension, most in countries with middle or low income (2). A global blood pressure screening study found that more than a third of people have high blood pressure and approximately a half are unaware they have it (3). Since high blood pressure rarely causes symptoms on its own, it is frequently disregarded. Hypertension, as a risk factor often leads to cerebral hemorrhage, heart failure, coronary heart disease and even illness (4). Recent studies also show that high blood pressure increases the incidence of breast cancer and Parkinson's disease (5, 6). Therefore, determining the best course of action for managing high blood pressure and reducing associated consequences has taken precedence.

The pathological mechanism of hypertension presents multi-dimensional interactive characteristics, involving multi-dimensional factors such as abnormal activation of the renin-angiotensin-aldosterone system (RAAS), imbalance of immune homeostasis, metabolic regulation disorders, vascular endothelial damage and genetic susceptibility (7, 8). Previous research (9) has connected the development of hypertension to oxidative stress, and the immune system plays a core regulatory role in it (8, 10). The evidence shows that hypertensive patients present with a persistent low-grade inflammatory state (11). The synergistic effect of innate and adaptive immune responses leads to abnormal activation and infiltration of monocytes/macrophages and lymphocytes, accompanied by enrichment of proinflammatory factors and chemokines, which aggravates oxidative stress. Oxidative stress reversely promotes the release of inflammatory mediators through signaling pathways (10). The two form a cascade amplification effect, jointly driving a vicious cycle of vascular remodeling and target organ damage. Besides, Oxidative stress occurs when there is an unbalanced concentration of oxidants and antioxidants in the body, with an emphasis on oxidants (12). This imbalance can cause disruptions to redox signaling, regulation, and molecular damage (12, 13). In addition to producing reactive oxygen species (ROS) by itself, which damages and kills endothelium, oxidative stress also interacts with the body's inflammatory proteins, hastening the death of cells (14, 15). Studies have shown that reducing oxidative stress in the body improves blood vessel stiffness and improves blood pressure (16).

However, several factors have a limited effect on the total oxidant/antioxidant system. Variations in dietary composition, obesity, exercise, smoking, and other lifestyle choices impact the body's degree of oxidative stress. Thus, we selected the oxidative balance score (OBS) as a measure to quantify the effect of diverse diets and lifestyles on the entire oxidant/antioxidant system (17). OBS is a composite index of 20 distinct dietary and lifestyle elements that accentuates the overall balance of antioxidants and oxidants at the dietary level. It's acknowledged that a higher OBS indicates superior antioxidant status. Previous researches have demonstrated an adverse association between OBS and various diseases, including metabolic syndrome (18), depression (19), breast cancer (20), and type 2 diabetes (21). Here, we explored the connection between OBS and the incidence of hypertension. We investigated the potential effects of OBS on hypertension using data from the National Health and Nutrition Examination Survey (NHANES) from 2007–2018.

## 2 Method

### 2.1 Extraction of data and study population

**Figure 1:**
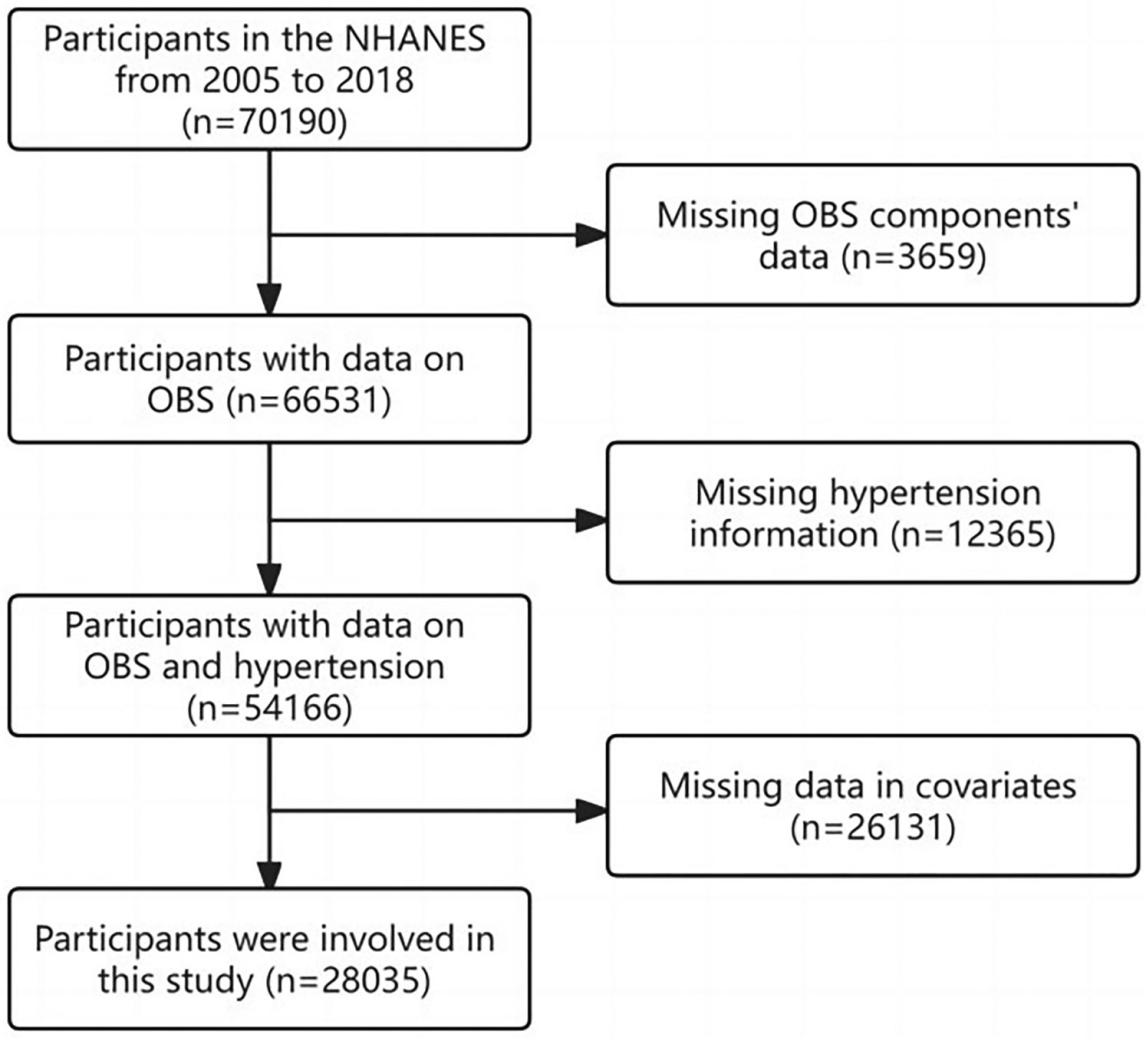
Flow chart.

NHANES employs a multistage, stratified probability approach to pick a representative sample of US citizens. It has been approved by the Ethics Review Board of the National Center for Health Statistics, and all participants have provided written, informed consent. It comprises of interview and examination components. In this study, data from seven NHANES cycles spanning from 2005 to 2006 to 2017–2018 were utilized, resulting in an initial pool of 70,190 patients. After excluding patients lacking dietary or lifestyle data, hypertension data, and variables with missing values, 28,035 subjects were included in the study (Figure 1).

### 2.2 Evaluation of diabetes (outcomes)

To determine whether our patients had hypertension, we employed three different techniques. First, the presence of hypertension was confirmed if it was indicated in the medications prescribed to the patient. Second, Questionnaires have been employed to gather information about hypertension. “Has a doctor or other health professional ever told you that you have high blood pressure, also known as hypertension?” was the question that was used to measure self-reported hypertension. Prior studies have also confirmed the use of self-reported hypertension (22). Third, we computed the patient's average blood pressure, either systolic or diastolic. Patients were classified as hypertensive if their systolic blood pressure was equal to or greater than 140 mm Hg, or if their diastolic blood pressure was equal to or greater than 90 mm Hg. Therefore, hypertension was considered an outcome variable in our study.

### 2.3 Calculation of the oxidative balance score (exposure)

Based on a previous study (23), OBS was determined by screening 16 nutrients and 4 lifestyle factors, encompassing 15 antioxidants and 5 pro-oxidants. There was a favorable correlation between participants' antioxidant activity and higher OBS. Dietary intake of 16 nutrients, including dietary fiber, carotene, riboflavin, niacin, vitamin B6, total folate, vitamin B12, vitamin C, vitamin E, calcium, magnesium, zinc, copper, selenium, total fat, and iron, was obtained from the first dietary review interview. The four lifestyle factors are physical activity, body mass index (BMI), alcohol consumption, and smoking. The amount of smoking is measured in terms of cotinine. The remaining factors were classified as antioxidants, while total fat, iron, BMI, alcohol use, and smoking were classified as pro-oxidants.

### 2.4 Covariates

We incorporated potential covariates that could impact the relationship between OBS and hypertension, such as sociodemographic variables, dietary quality, lipid-related indicators, lifestyle, and co-morbidities. Sociodemographic variables encompassed age, gender (male/female), race (black, Mexican, white, and other), education (<high school, high school, college), BMI, and poverty income ratio (PIR). Total calorie intake and the 2015 Healthy Eating Index (HEI) have been employed to judge overall dietary quality (24). Total cholesterol, and triglycerides are examples of lipid-related markers. Lifestyle primarily involved smoking and alcohol consumption. Co-morbidities included diabetes and hyperlipidemia. Furthermore, we employed stratified variables for age (below/above 60), sex (male/female), hyperlipidemia (yes/no), diabetes (DM, IFG, IGT, NO), and pre-specified effect modifiers to assess the interaction effect.

### 2.5 Statistical analysis

Due to the complexity of stratified sampling, weighted statistics are used in this paper. We applied the frequency (percentage) for representation and chi-square tests for categorical variables and the mean (standard deviation) for representation and *t*-tests for continuous variables. To validate the correlation between OBS and hypertension and explore the potential nonlinear relationship, OBS was converted from a continuous variable into a categorical variable by utilizing quartiles (Q1, Q2, Q3, and Q4), and the *P*-value for trend was determined.

Multivariable logistic regression models (from crude model to model 2) were utilized to examine the relationship between OBS and hypertension while adjusting for various potential confounders. The only covariate in the crude model that was changed was OBSQ. Model 1 was corrected for age, gender, ethnicity, and OBSQ. Model 2 further adjusted for education, PIR, BMI, hyperlipidemia, DM, kcal, HEI, smoke, triglycerides, total cholesterol, and HDL. Heterogeneity between OBS and hypertension was further assessed through subgroup analysis based on variables such as age groups, gender, hyperlipidemia, and diabetes. Smooth curves were also plotted to display the association between OBS and hypertension.

All statistical analyses were conducted using R software (version 4.2) or Empowerstats (version 4.1). A significance threshold of alpha <0.05 was set, and all analyses were two-sided. A *p*-value of 0.05 or less on both sides was deemed statistically significant.

## 3 Results

### 3.1 Baseline characteristics

The study reported 28,035 samples in total, with a median age of 47.03, and 41.72% of those samples had hypertension (Table 1). The distribution of genders was fairly equal. The majority of the participants (12,662, 45.16%) were white, followed by black (5,687, 20.29%), individuals of other races (5,349, 19.08%), and Mexican individuals (4,337, 15.47%). The proportion of highly educated individuals in the population is greater than 50% (*n* = 14,939, 53.29%). Participants had lower BMI and smoking rates, but higher age, PIR, calorie, HEI, and HLD values, as well as a higher percentage of men and higher levels of education, as compared to the lowest OBS quartile.

**Table 1:** The distribution of baseline characteristics by quartiles of the OBS, national health and nutrition examination survey 2005–2018.

Characteristic	Total *n* = 28,035	Q1 (0,12) *n* = 7,358	Q2 (12,19) *n* = 7,028	Q3 (19,25) *n* = 6,863	Q4 (25,37) *n* = 6,786	*p*-value
Age	47.03 (0.26)	45.07 (0.33)	48.21 (0.34)	47.44 (0.33)	47.29 (0.40)	<0.0001
PIR	3.03 (0.03)	2.56 (0.04)	2.89 (0.04)	3.14 (0.04)	3.44 (0.04)	<0.0001
BMI	29.01 (0.09)	29.59 (0.12)	29.69 (0.13)	29.31 (0.13)	27.71 (0.12)	<0.0001
Total calorie intake	2,167.05 (8.90)	1,815.30 (17.09)	1,862.32 (13.64)	2,205.96 (14.68)	2,666.99 (19.53)	<0.0001
Healthy eating index	50.88 (0.23)	45.85 (0.30)	47.87 (0.24)	51.20 (0.24)	57.14 (0.30)	<0.0001
Triglycerides	155.15 (1.27)	156.05 (1.82)	160.95 (2.42)	158.07 (2.45)	147.02 (1.88)	< 0.0001
Total cholesterol	195.23 (0.50)	193.63 (0.68)	196.35 (0.86)	195.35 (0.78)	195.47 (0.86)	0.03
HDL	53.37 (0.21)	51.55 (0.34)	52.39 (0.28)	53.29 (0.33)	55.72 (0.30)	<0.0001
Sex						<0.001
Female	13,970 (49.83)	3,336 (47.65)	3,558 (51.29)	3,588 (53.29)	3,488 (50.63)	
Male	14,065 (50.17)	4,022 (52.35)	3,470 (48.71)	3,275 (46.71)	3,298 (49.37)	
Ethnic						<0.0001
Black	5,687 (20.29)	1,950 (15.19)	1,642 (12.44)	1,218 (8.88)	877 (5.62)	
Mexican	4,337 (15.47)	1,162 (9.37)	1,054 (8.25)	1,084 (8.27)	1,037 (7.27)	
Other	5,349 (19.08)	1,374 (13.31)	1,253 (11.63)	1,309 (11.69)	1,413 (11.79)	
White	12,662 (45.16)	2,872 (62.13)	3,079 (67.67)	3,252 (71.16)	3,459 (75.32)	
Education						<0.0001
College and high	14,939 (53.29)	3,004 (49.34)	3,467 (56.21)	3,926 (63.47)	4,542 (74.52)	
High school	6,574 (23.45)	1,978 (28.60)	1,763 (26.70)	1,577 (23.75)	1,256 (16.97)	
Less than high school	6,522 (23.26)	2,376 (22.06)	1,798 (17.09)	1,360 (12.77)	988 (8.52)	
Hyperlipidemia						<0.0001
No	8,117 (28.95)	2,084 (29.12)	1,858 (26.00)	1,915 (28.07)	2,260 (33.24)	
Yes	19,918 (71.05)	5,274 (70.88)	5,170 (74.00)	4,948 (71.93)	4,526 (66.76)	
Diabetes						<0.0001
DM	5,092 (18.16)	1,563 (15.86)	1,461 (16.51)	1,191 (13.65)	877 (9.90)	
IFG	1,300 (4.64)	358 (4.77)	338 (4.66)	330 (4.83)	274 (3.81)	
IGT	1,126 (4.02)	281 (3.15)	285 (3.60)	297 (3.94)	263 (3.29)	
No	20,517 (73.18)	5,156 (76.22)	4,944 (75.23)	5,045 (77.58)	5,372 (83.00)	
Smoke						<0.0001
Former	6,822 (24.33)	1,610 (20.52)	1,767 (25.50)	1,725 (25.53)	1,720 (26.67)	
Never	15,456 (55.13)	3,544 (46.88)	3,730 (51.48)	3,890 (56.87)	4,292 (62.72)	
Now	5,757 (20.54)	2,204 (32.60)	1,531 (23.02)	1,248 (17.60)	774 (10.61)	
Alcohol						<0.0001
Former	4,513 (16.1)	1,324 (14.95)	1,262 (14.32)	989 (11.53)	938 (11.99)	
Heavy	5,750 (20.51)	1,735 (27.49)	1,419 (23.07)	1,390 (21.24)	1,206 (17.48)	
Mild	9,438 (33.67)	2,062 (28.55)	2,320 (35.44)	2,395 (36.64)	2,661 (43.44)	
Moderate	4,392 (15.67)	1,106 (17.26)	1,016 (16.20)	1,159 (19.29)	1,111 (18.00)	
Never	3,942 (14.06)	1,131 (11.74)	1,011 (10.98)	930 (11.30)	870 (9.09)	
Hypertension						<0.0001
No	16,338(58.28)	4,097(61.10)	3,798(58.33)	4,070(62.16)	4,373(67.22)	
Yes	11,697(41.72)	3,261(38.90)	3,230(41.67)	2,793(37.84)	2,413(32.78)	

Mean (SD) for continuous variables: the *P-*value was calculated by the t-tests. Frequency (percentage) for categorical variables: the *P-*value was calculated by the chi-square tests. Q, quartitle; PIR, ratio of family income to poverty; BMI, body mass index; DM, diabetes; IFG, impaired fasting glucose; IGT, impaired glucose tolerance.

### 3.2 Association between OBS and hypertension

**Figure 2:**
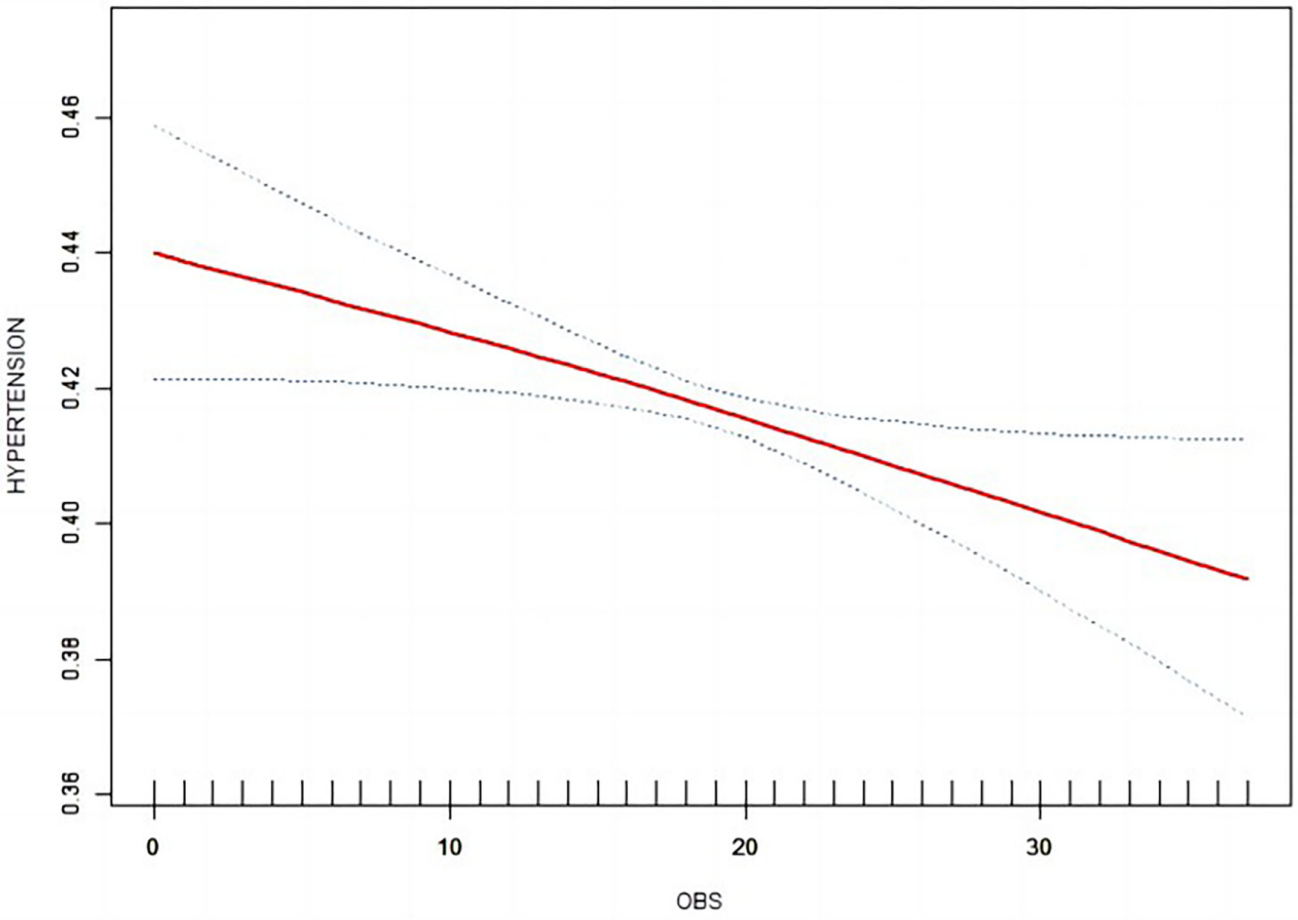
Smooth curve between OBS and hypertension.

Weighted logistic regression analysis revealed a correlation between OBS and hypertension, as indicated in Table 2. Irrespective of covariate adjustments, a substantial inverse correlation was noted between the highest OBS quartile and the lowest OBS quartile with the likelihood of developing hypertension. In the crude model, however, there was a positive association between the second quartile of OBS and hypertension. Further sensitivity analysis confirmed this pattern (*p* for trend < 0.0001). Smooth curve (Figure 2) fitting revealed a declining probability of hypertension occurrence with increasing OBS values, consistent with the logistic regression outcomes. Additionally, after taking the logarithm of the odds ratio for hypertension, it was evident that higher OBS values were associated with a reduced risk of developing hypertension.

**Table 2:** Weighted logistic regression analysis models showing the relationship between OBS and hypertension.

nhs—Hypertension	OBSQ					
	Crude model		Model 1		Model 2	
Character	95% CI	*P*	95% CI	*P*	95% CI	*P*
Q1	ref		ref		ref	
Q2	1.12 (1.03,1.23)	0.01	0.95 (0.86,1.06)	0.34	0.97 (0.87,1.09)	0.61
Q3	0.96 (0.86,1.06)	0.39	0.85 (0.76,0.95)	0.003	0.90 (0.79,1.01)	0.08
Q4	0.77 (0.69,0.85)	<0.0001	0.67 (0.60,0.75)	<0.0001	0.81 (0.70,0.93)	0.003
*p* for trend (character2 integer)		<0.0001		<0.0001		0.002
*p* for trend (Median value)		<0.0001		<0.0001		0.002

Crude model: OBSQ; Model 1: OBSQ, age, sex, ethnic; Model 2: OBSQ, age, sex, ethnic, education, ratio of family income to poverty, body mass index, hyperlipidemia, diabetes, total calorie intake, healthy eating index, smoke, triglycerides, total cholesterol, HDL. The specific range for the quantiles is consistent with Table 1.

### 3.3 Subgroup analysis

Table 3 displays the results of the subgroup analysis split down by age, gender, diabetes, and hyperlipidemia. The age subgroup of hypertension showed statistical significance (*p* for interaction = 0.041). Within the age subgroup, individuals under 60 years old were found to be more responsive to higher OBS compared to those over 60. In other words, the population of lower 60 years had an 17.9% lower risk of developing hypertension in the top quartile compared with the first quartile (all *p* for trend <0.05). Men in the fourth quartile of the OBS population who do not have diabetes mellitus or concomitant hyperlipidemia can also be observed to have a strong negative connection with hypertension.

**Table 3:** Subgroup analysis.

Characteristic	Q1 (0,12) *n* = 7,358	Q2 (12,19) *n* = 7,028	*p*	Q3 (19,25) *n* = 6,863	*p*	Q4 (25,37) *n* = 6,786	*p*	*p* for trend (character2 integer)	*p* for trend (Median value)	*p* for interaction
Age										0.041
Age < 60	Ref	0.978 (0.859,1.113)	0.734	0.932 (0.810,1.073)	0.322	0.821 (0.693,0.972)	0.022	0.026	0.026	
Age>=60	Ref	1.138 (0.944,1.371)	0.173	0.994 (0.824,1.197)	0.945	0.966 (0.769,1.213)	0.762	0.461	0.565	
Sex										0.581
1	Ref	1.008 (0.869,1.168)	0.916	0.951 (0.806,1.121)	0.544	0.783 (0.667,0.919)	0.003	0.004	0.005	
0	Ref	1.159 (0.988,1.359)	0.070	1.029 (0.879,1.204)	0.719	0.990 (0.820,1.195)	0.914	0.641	0.799	
Hyperlipidemia										0.135
Yes	Ref	1.088 (0.965,1.227)	0.167	0.991 (0.882,1.115)	0.883	0.873 (0.756,1.007)	0.062	0.039	0.06	
No	Ref	0.957 (0.763,1.201)	0.702	0.778 (0.622,0.972)	0.027	0.677 (0.531,0.864)	0.002	<0.001	<0.001	
DM										0.428
No	Ref	1.058 (0.937,1.195)	0.361	0.940 (0.825,1.072)	0.352	0.805 (0.699,0.926)	0.003	0.001	0.002	
DM	Ref	1.047 (0.794,1.380)	0.743	0.904 (0.696,1.175)	0.448	1.023 (0.685,1.527)	0.911	0.823	0.859	
IFG	Ref	1.384 (0.836,2.288)	0.203	1.162 (0.676,1.999)	0.582	1.090 (0.625,1.902)	0.758	0.823	0.73	
IGT	Ref	0.918 (0.554,1.522)	0.738	0.948 (0.561,1.601)	0.840	0.994 (0.587,1.682)	0.981	0.967	0.999	

DM, diabetes; IFG, impaired fasting glucose; IGT, impaired glucose tolerance.

## 4 Discussion

28,035 samples from seven NHANES cycles between 2005 and 2018 have been included in this study's analysis of the connection between OBS and hypertension. We discovered in this study that OBS was considerably higher in non-hypertension individuals than in hypertensive individuals. Additionally, a higher OBS demonstrated a negative relationship with the likelihood of developing hypertension. Upon accounting for potential influencing factors, the findings indicated that the impact of OBS on hypertension was significantly influenced by age. People under 60 were more likely to benefit from the trial and had a lower risk of developing hypertension than people over 60.

Numerous studies on animals and in humans have tied oxidative stress to the onset and development of hypertension. When the quantity of ROS surpasses the antioxidants' ability to scavenge them, oxidative stress results. In addition to directly affecting cellular macromolecules, which may result in cell damage and death (25), oxidative stress also influences inflammatory factors, which can accelerate blood vessel aging and elevate blood pressure (15). The probability of cardiovascular disease climbs with age, indicating that age is an important factor in cardiovascular disease (26). A cohort study revealed a significant link between age and hypertension, particularly hastening the onset of hypertension in middle age (27). In aged hypertensive mice, Angela Wirth et al. (28) discovered the development of accelerated endothelial cell dysfunction in conjunction with elevated ROS in the vasculature. Besides, as we age, blood vessels begin to stiffen, and when the endothelial cells in the blood vessels are attacked by oxidants or inflammatory substances, the blood vessels stiffen rapidly, increasing blood flow (29). Aged cardiomyocytes have been shown to have increased free radicals and a chronic proinflammatory state too (30). The middle intima of medium-sized arteries thickens as a result of endothelial cell failure brought on by high levels of oxidative stress and inflammation *in vivo* (31). The renewal rate of cardiomyocytes is highest at around 20 years of age, declining steadily thereafter to less than 0.5% per year in the elderly (32). Based on these findings, it is believed that younger patients have better vascular conditions and are more likely to benefit from antioxidants.

It has been proven that the NOX family plays a substantial part in the development of hypertension (33). The production of oxides by the NOX family during the redox process in the population is the main reason for the increase in blood pressure (34, 35). In experiments with hypertensive mice, blocking the NOX pathway with a drug led to an improvement in blood vessel function and normalization of blood pressure (36). Consequently, NOX isoforms (NOX2) may be expressed in immune cells, producing high levels of oxygen free radicals and causing damage to the vascular endothelium (37).

Several research have also verified the positive correlation between antioxidants and a decreased risk of hypertension. A retrospective study discovered that eating foods high in antioxidants, such as lycopene, α-carotene, β-carotene, lutein with zeaxanthin, and total carotenoids, reduced the incidence of high blood pressure (38). Besides, in a dietary study aimed at elderly patients with metabolic syndrome (MetS) in Korea, when patients ate foods rich in antioxidants for 4 weeks, changes in the state of oxidative stress and MetS involving hypertension, arteriosclerosis and dyslipidemia (39). Additionally, *in vitro* experiments conducted on mice demonstrated that resveratrol plays a role in lowering blood pressure, improving oxidative stress, and enhancing cellular endothelial function (40). Furthermore, a recent study on mice demonstrated that raising the levels of the mitochondrial deacetylase sirt3 decreased oxidative stress, prevented endothelial dysfunction and vascular inflammation, and slowed down hypertension and vascular aging (41). Patients with hypertension who received the physiological oxidant melatonin1 for that year exhibited improvements in arterial stiffness and a drop in serum TAC (a measure of oxidative stress), according to randomized controlled research (42).

According to this research, an antioxidant-rich diet and way of life are good for mental health. In addition, there was age dimorphism in these trends, with younger people seeing a higher protective impact from antioxidant diet and lifestyle choices. The results emphasize the need of maintaining a diet and way of life high in antioxidants, as they can aid in the prevention and treatment of hypertension.

This study revealed the dual value of antioxidant dietary patterns and lifestyle interventions in the management of hypertension. Data from a cohort study confirmed that higher OBS in hypertensive patients was significantly associated with reduced all-cause mortality (43). Coincidentally, a large-scale cross-sectional study conducted in China also found that a healthy lifestyle and a diet rich in antioxidants can prevent hypertension (44). These findings reinforce the pivotal role of the balance of the oxidation/antioxidant system in blood pressure regulation from an epidemiological perspective. It is worth noting that OBS, as a quantitative assessment tool, can provide individuals with a visual oxidative stress risk assessment by integrating dietary components and lifestyle parameters, thereby guiding precise nutritional counseling.

Our research advantages lie in the following aspects. First, we used seven waves of high-quality, representative data from the NHANES, which are large and multi-stage. Second, the logistic regression model was adjusted to eliminate some potential confounding factors, including demographic, socioeconomic, health and lifestyle information, to obtain more accurate results.

There are a few other restrictions on the current investigation. First, because the study is cross-sectional, it is challenging to determine a causal association between OBS and hypertension. Thus, more research with a prospective design is required to demonstrate the effectiveness of OBS. In the future, prospective study designs will be used to track the association between dynamic changes in OBS and blood pressure trajectories and to use bidirectional Mendelian methods to explore the causal relationship between OBS and hypertension. Second, it is difficult to integrate all OS-related nutrition and lifestyle exposures into OBS; flavonoids, for example, were one of the components with restricted availability. Subsequent studies need to integrate food composition databases to more accurately quantify dietary antioxidant exposure. Third, all pro-oxidants and antioxidants are assumed to have a linear correlation with oxidative stress, ignoring the threshold effects of antioxidants. Finally, the limited data did not account for the effect of hypertensive medications on hypertensive patients, making it impossible to determine whether there was a differential effect of OBS between hypertensive patients taking or not taking hypertensive medications. It is anticipated that this problem will be addressed in later clinical research.

## 5 Conclusion

In conclusion, our analysis suggested that there was a substantial reverse correlation among OBS and hypertension. The study revealed that higher OBS, which signifies greater antioxidant exposure compared to prooxidant exposure in diet and lifestyle, is linked to decreased likelihood of hypertension. The precise mechanism behind the association between OBS and hypertension as well as its causal link warrant further investigation.

## Data Availability

Publicly available datasets were analyzed in this study. This data can be found here: https://www.cdc.gov/nchs/nhanes/index.htm.
